# Perforated sigmoid colon in the setting of chicken bone ingestion and diverticulitis: A case report

**DOI:** 10.1016/j.amsu.2021.102650

**Published:** 2021-07-30

**Authors:** Karthik Kanamalla, Frank J. Salamone, Jose Vargas

**Affiliations:** a Frank H. Netter MD School of Medicine, Quinnipiac University, 370 Bassett Rd, North Haven, CT 06473, USA; b St. Vincent's Medical Center, 2800 Main St, Bridgeport, CT 06606, USA

**Keywords:** Diverticulitis, Perforation, Chicken bone, Foreign body, Hinchey classification, Case report

## Abstract

**Introduction:**

Diverticular perforation due to foreign body ingestion is an uncommon but important cause of gastrointestinal tract injury. The aim of this study is to discuss relevant findings seen in diverticulitis caused by foreign bodies and its treatment.

**Case presentation:**

In this report, we present a case of a 30-year-old woman who presented to the emergency department with two days of severe abdominal pain and diarrhea. Computed tomography of the patient's abdomen and pelvis revealed micro-perforations of the sigmoid colon with pneumoperitoneum and an intraluminal foreign body. She subsequently underwent an exploratory laparotomy with sigmoid resection and end-to-end anastomosis due to acute diverticulitis complicated by feculent peritonitis. Gross examination of the excised specimen revealed two large perforations and an intraluminal chicken bone. After a six-day hospitalization, the patient was discharged with an excellent prognosis.

**Discussion and conclusion:**

Prompt radiological evaluation and classification of the degree of diverticulitis using the Hinchey classification system in this patient helped guide definitive treatment. Usage of this classification scheme in foreign body diverticulitis is valuable in determining whether a surgical or non-surgical approach is necessary.

## Highlights

• Foreign bodies, such as chicken bones, are known causes of diverticular perforation.
• Computed tomography is the best test in locating foreign bodies and complications.
• The Hinchey classification informs necessity of surgical or non-surgical treatment.

## 1 Introduction

Diverticular disease is a largely asymptomatic condition resulting from herniation of colonic mucosa and submucosa through the bowel wall at sites where the vasa recta perforates the muscularis layer. The cause of diverticulum formation is multifactorial and includes genetics, dietary influences, increased age, and even the gut microbiome [1]. Although a significant proportion of adults have diverticulosis, it is estimated that approximately 4% of these individuals will develop diverticular inflammation, or diverticulitis, over their lifetime [2]. Complications of diverticulitis include obstruction, abscesses, fistulas, and perforation. It is hypothesized that overgrowth of intestinal flora within a diverticulum contributes to the development of diverticulitis; however, some studies illustrate that environmental factors and host immune dysregulation also play a role as resolution of diverticulitis without antibiotics has been observed in some patients [3]. Rare cases of diverticulitis secondary to intraluminal foreign bodies have also been described in the literature. In this report, we present a case of feculent sigmoid diverticulitis secondary to accidental ingestion of a chicken bone.

This case report has been reported in line with the SCARE Criteria [4].

## 2 Case presentation

**Fig. 1:**
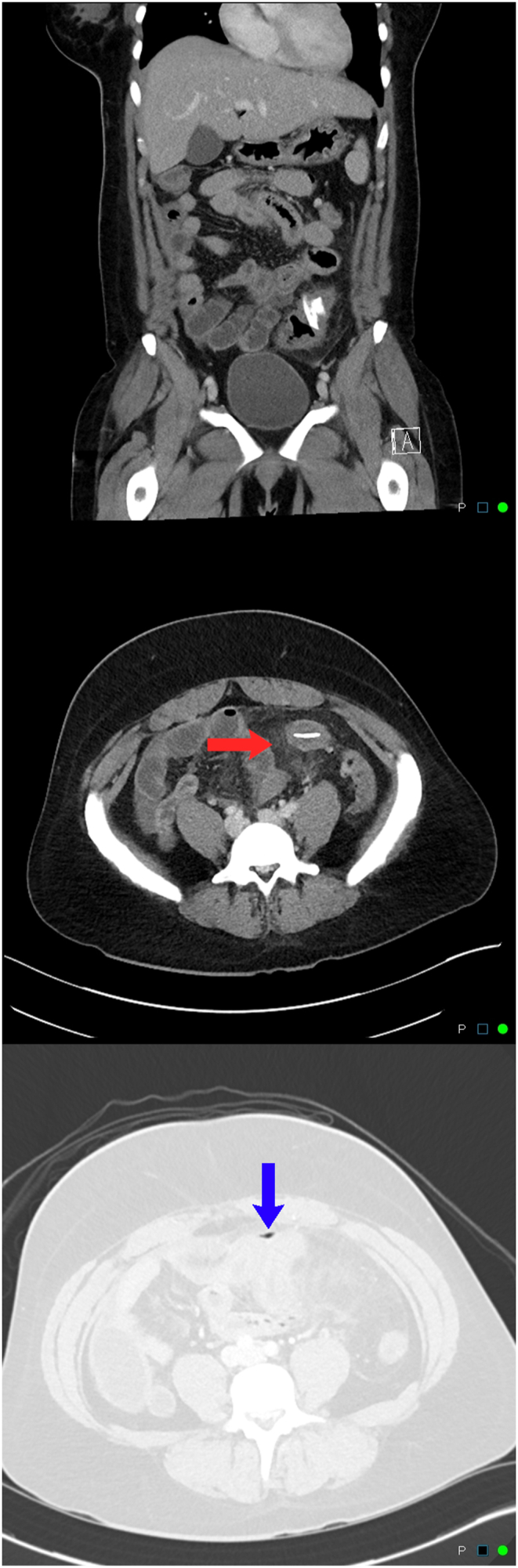
Coronal (top) and axial (middle and bottom) sections of the abdomen and pelvis are shown depicting a hyperdense intraluminal sigmoid foreign body and perisigmoid fat stranding (red arrow). Intraperitoneal air foci are also shown (blue arrow). (For interpretation of the references to colour in this figure legend, the reader is referred to the Web version of this article.)

A previously healthy 30-year-old female security guard with no past surgical or medical history self-presented to the emergency department from work for evaluation of a two-day history of severe, cramping periumbilical abdominal pain and diarrhea. During this time, she had also had chills, nausea, and cough. The patient was in significant distress and discomfort due to her pain and her physical exam revealed tachycardia, hypertension, and diffuse abdominal tenderness to palpation with rebound. Complete blood count and liver function tests showed a white blood cell count of 18,800/mm^3^ with 92.9% segmented neutrophils and a total bilirubin of 2.5 mg/dL (direct 0.9 mg/dL), respectively. Urinalysis was unremarkable and urine β-hCG was negative. At this point, the differential diagnosis included appendicitis, acute colitis, and gastroenteritis. Computed tomography (CT) of the abdomen and pelvis with intravenous contrast revealed perisigmoid fat-stranding and multiple air foci within the peritoneum. A linear density was also appreciated within the sigmoid colon (Fig. 1). These findings likely represented colonic micro-perforation secondary to a foreign body.

**Fig. 2:**
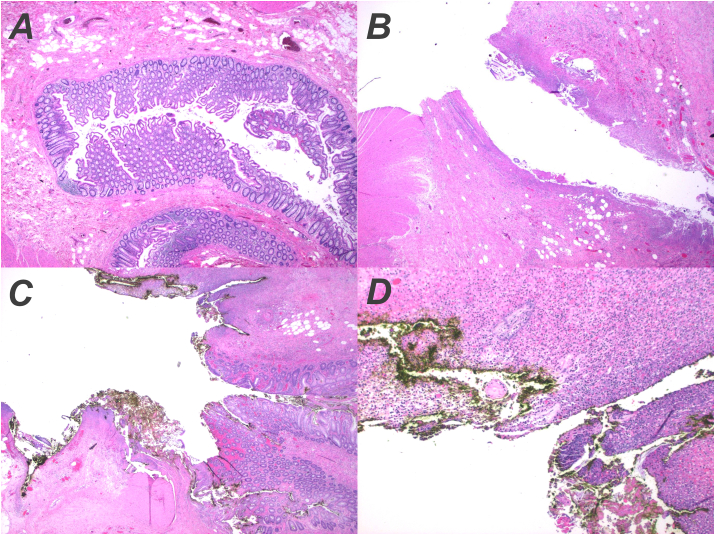
Histological preparation of surgical specimen (hematoxylin and eosin stain) illustrating a diverticulum characterized by outpouching of mucosal tissue into the bowel wall (A), as well as a large (B), and two smaller (C and D) perforations. (For interpretation of the references to colour in this figure legend, the reader is referred to the Web version of this article.)

The patient was immediately taken to the operating room for an exploratory laparoscopy under general anesthesia; however, this was not possible as liquid stool was present in the peritoneal cavity. She subsequently underwent an exploratory laparotomy with sigmoidectomy which was performed by two attending trauma surgeons and a physician assistant. The decision was made to perform an end-to-end anastomosis instead of a colostomy as the anterior abdominal wall was over four inches thick and some difficulty was encountered with mobilization of the descending colon. In addition, the patient had a low diverticular burden with a healthy and well-vascularized distal margin, supporting the decision for primary anastomosis. Intraoperative findings confirmed Hinchey class IV sigmoid diverticulitis. After placement of a 19 French Blake drain within the pelvis, the patient was extubated and transferred to the postanesthesia care unit without any intraoperative complications. Total operative time was approximately 5 h. She was then taken to the surgical floor where she received piperacillin/tazobactam, and pain and nausea management with morphine and ondansetron. Bowel function returned on postoperative day three and diet and activity were advanced as tolerated. After an uneventful six-day hospitalization, she was discharged with oral levofloxacin and metronidazole to be taken at home for three days. She was followed up on an outpatient basis for wound care and recovered satisfactorily. Pathologic examination of the resected colon showed two areas of perforation measuring approximately 0.5 cm and 1.1 cm, respectively, in diameter. A diverticulum was also seen within the specimen with inflammatory changes. Finally, a fragment of a yellow-tan, firm cortical bone measuring 3.9 × 2.1 × 0.2 cm was retrieved from the lumen. Histological findings are shown in Fig. 2.

## 3 Discussion

Gastrointestinal (GI) tract perforation may arise due to a multitude of etiologies. The stomach and duodenum are the most frequently involved sites. Causes of perforation in these areas include peptic ulcer disease and ulceration of malignancies (i.e. carcinomas or gastric lymphomas) [5]. Perforation of the mesenteric small bowel is rare and accounts for only 0.4% of all cases of GI tract perforation [6]. Crohn's disease, diverticulitis, ischemia, bevacizumab therapy, and colonoscopy are known causes of large bowel perforation. It is estimated that less than 1% of ingested foreign bodies cause GI tract perforation [7]. However, coexisting diverticulitis in this patient may have increased the risk of perforation. Most often, foreign objects pass through the alimentary canal uneventfully, but can become lodged in the esophagus, stenotic regions, or areas of anatomic angulation (i.e. duodenal loop, duodenojejunal junction, and the appendix) [8]. In the large intestine, the most common areas for foreign bodies to cause complications are in the ileocecal and rectosigmoid regions [8]. Although children and those with psychiatric conditions or intellectual disability may intentionally ingest inedible objects, most patients who are found to have GI foreign bodies do not recall consuming the offending agent – as was the case with the patient described above [9].

In one literature review of diverticulitis caused by foreign bodies, objects included toothpicks, fish bones, biliary stents, wooden skewers, plastic bag clips, a blister-wrapped tablet, a metal staple, and a vegetable stalk [10]. From the 50 case reports included in this review, 21 were caused by ingestion of chicken bones of which eight were complicated by perforation. In these cases, a diagnosis was established with abdominopelvic CT scan, upright x-ray and laparotomy, or colonoscopy. Abdominal x-ray is unlikely to accurately detect a foreign body unless it is composed of high-density bone or metal [11].

In current practice, if the history and physical exam are concerning for perforated diverticulitis, cross-sectional imaging should be utilized to confirm the diagnosis. Radiographic findings include intra-abdominal free air, focal bowel wall discontinuity, bowel wall thickening, inflammatory fat stranding, extravasation of oral contrast, and intra-abdominal fluid collections or abscesses [5]. Identification of the site and cause of perforation on abdominopelvic CT scan is aided when performed with and without administration of intravenous contrast medium as this allows for the detection of indirect signs of bowel perforation, such as bowel wall abnormalities [12]. Although extravasation of oral contrast is a reliable direct sign, it may impair detection of a radio-opaque foreign body and delay diagnosis in the emergency setting [13].

Management of sigmoid diverticulitis due to a foreign body is determined based on which complications are present. In 1978, Hinchey et al. proposed a grading system characterizing the extent and nature of complicated diverticular disease [14]. This classification system is summarized in Table 1. Hartmann's procedure is indicated for patients with Hinchey class IV diverticulitis or patients with hemodynamic instability (even in Hinchey class II diverticulitis) [15]. However, this procedure is associated with higher morbidity and mortality when compared to primary anastomosis [16]. In young and otherwise healthy patients, such as the one reported here, sigmoidectomy with primary anastomosis may be associated with better outcomes [17]. All cases of perforated (Hinchey class III or IV) diverticulitis due to chicken bone ingestion mentioned in the literature were managed surgically [10]. Selected procedures included subtotal colectomy with ileorectal anastomosis and Hartmann's procedure. In one case, laparoscopic removal of the bone with peritoneal washout and drain was used successfully. Utility of this technique in cases of perforated diverticulitis is best determined on a patient-by-patient basis [18]. In certain cases of uncomplicated or non-perforated diverticulitis, colonoscopic retrieval of the foreign body was performed [10]. This case is particularly unique in that it demonstrates the utility of sigmoid resection with end-to-end anastomosis in perforated foreign body diverticulitis.

**Table 1:** Hinchey classification.

I	Pericolic abscess or phlegmon
II	Pelvic, intraabdominal, or retroperitoneal abscess
III	Generalized purulent peritonitis
IV	Generalized fecal peritonitis

## 4 Conclusions

Foreign body ingestion is a rare but important cause of diverticular perforation. Early cross-sectional imaging in patients presenting with signs and symptoms of acute diverticulitis may reveal representative radiographic findings and intraluminal foreign objects. Accurate determination of the extent of diverticulitis and identification of complications using the Hinchey classification system is useful in guiding treatment and possibly averting the need for surgical intervention in favor of minimally invasive options like colonoscopic foreign body removal.

## Ethical approval

None.

## Funding

None.

## Author contribution

K.K. and F.J.S. were responsible for the writing of this paper. J.V. was responsible for data analysis, interpretation, and critical review of the draft.

## Registration of research studies

Name of the registry: n/a

Unique Identifying number or registration ID: n/a

Hyperlink to your specific registration (must be publicly accessible and will be checked): n/a Hyperlink to your specific registration (must be publicly accessible and will be checked): n/a

## Guarantor

Jose Vargas, M.D. (josevargasmd@gmail.com)

## Patient consent

Written informed consent was obtained from the patient for publication of this case report and accompanying images. A copy of the written consent is available for review by the Editor-in-Chief of this journal on request.

## Provenance and peer review

Not commissioned, externally peer-reviewed.

## Declaration of competing interest

The authors of this study have no conflicts of interest to disclose.
